# Light Processable Starch Hydrogels

**DOI:** 10.3390/polym12061359

**Published:** 2020-06-17

**Authors:** Camilla Noè, Chiara Tonda-Turo, Annalisa Chiappone, Marco Sangermano, Minna Hakkarainen

**Affiliations:** 1 Politecnico di Torino, Dipartimento di Scienza Applicata e Tecnologia C.so Duca Degli Abruzzi 24, 10129 Torino, Italy; camilla.noe@polito.it (C.N.); chiara.tondaturo@polito.it (C.T.-T.); annalisa.chiappone@polito.it (A.C.); marco.sangermano@polito.it (M.S.); 2 Department of Fibre and Polymer Technology, KTH Royal Institute of Technology, 100 44 Stockholm, Sweden

**Keywords:** hydrogel, starch, UV curing, 3D printing, digital light processing

## Abstract

Light processable hydrogels were successfully fabricated by utilizing maize starch as raw material. To render light processability, starch was gelatinized and methacrylated by simple reaction with methacrylic anhydride. The methacrylated starch was then evaluated for its photocuring reactivity and 3D printability by digital light processing (DLP). Hydrogels with good mechanical properties and biocompatibility were obtained by direct curing from aqueous solution containing lithium phenyl-2,4,6-trimethylbenzoylphosphinate (LAP) as photo-initiator. The properties of the hydrogels were tunable by simply changing the concentration of starch in water. Photo-rheology showed that the formulations with 10 or 15 wt% starch started curing immediately and reached G’ plateau after only 60 s, while it took 90 s for the 5 wt% formulation. The properties of the photocured hydrogels were further characterized by rheology, compressive tests, and swelling experiments. Increasing the starch content from 10 to 15 wt% increased the compressive stiffness from 13 to 20 kPa. This covers the stiffness of different body tissues giving promise for the use of the hydrogels in tissue engineering applications. Good cell viability with human fibroblast cells was confirmed for all three starch hydrogel formulations indicating no negative effects from the methacrylation or photo-crosslinking reaction. Finally, the light processability of methacrylated starch by digital light processing (DLP) 3D printing directly from aqueous solution was successfully demonstrated. Altogether the results are promising for future application of the hydrogels in tissue engineering and as cell carriers

## 1. Introduction

Hydrogels are three-dimensional polymeric networks with the ability of retaining large amount of water within their structures without dissolving. Their crosslinked networks can be formed by either chemical or physical gelation process. The physical gel process involves weak Van der Waals forces, electrostatic interaction, and hydrogen bonding while the chemical gel formation leads to strong chemically bonded networks. Thanks to their hydrophilicity, hydrogels can be utilized by a broad range of sectors including agro-industry, pharmaceutical, medical, and food applications [1,2,3,4,5]. Biopolymer (e.g., polysaccharide and protein) based hydrogels are gaining increasing attention, due to many favorable properties, such as being renewable and bacteriologically degradable [6,7,8]. Starch is an abundant polysaccharide composed of a mixture of two different D-glucose polymers: amylose and amylopectin. Amylopectin is a highly branched polymer, while amylose is linear. In nature, the extracellular matrix (ECM) of mammals is composed of a mixture of polysaccharides, water, soluble factors for signaling and cell-adhesive proteins. Starch based-hydrogels are therefore, partially able to mimic the natural cells aqueous environment, and this is very important when the hydrogel is to be used in tissue-engineering applications [9]. Starch-based hydrogels have been fabricated from water-starch solutions by applying ultrahigh pressure (UHP) at different temperatures [10] or by crosslinking with different types of crosslinkers such epichlorohydrin [11] or ammonium zirconium carbonate [12].

Photocuring techniques are green and highly efficient for crosslinking polymer resin with photo-curable functional groups. They have many advantages with respect to the thermocuring processes such as absence of Volatile Organic Compound (VOC) emissions, high curing speed, reduced energy consumption, and no heat requirements [13,14,15]. Recently UV curable biopolymer-resins have gained increasing interest [16,17]. For example, cationic UV-curable starch-based coatings were created by grafting of glycidyl methacrylate (GMA) onto the starch structure [18]. Starch-based hydrogel were also prepared by grafting vinyl monomers or by introducing other photocurable reactive groups along the starch chain [19,20,21,22]. Chemically crosslinked starch materials showed superior mechanical, chemical and thermal resistance over the non-crosslinked native starch [23]. Biodegradable films cured under UV-radiation were obtained by sago starch mixed in different concentration with PVA [24] or from UV-cured chitosan-starch films soaked in methyl methacrylate solutions [25]. Innovative techniques were also developed concerning the starch crosslinking in the solid state with either different photosensitizers [26] or with sodium benzoate as photoinitiator [27].

Starch is an excellent precursor for preparation of hydrogels. GMA was utilized to methacrylate starch through either a transesterification reaction or by epoxy ring-opening mechanism [28,29]. Starch has also been acrylated with acryloyl chloride, but to obtain a photocurable hydrogels the materials were subsequently modified with the zwitterionic monomer 3-dimethyl(methacryloyloxyethyl) ammonium propane sulfonate [30]. Hydroxyethyl starch functionalized with polyethylene glycol methacrylate was employed to obtain microspheres via emulsion polymerization [31,32]. Starch was also successfully modified with photocurable side groups using 4-pentenoic anhydride yielding starch-pentenoate (SP). The material was subsequently cured with a bifunctional thiol-crosslinker. However, in this work, the starch hydrogels without additives were not robust enough to be manipulated [9,33].

3D printing by fused filament fabrication (FFF) has gained significant attention as a tool for designing biomedical scaffolds [34]. More recently combination of photo-curable resins with rapid prototyping photo-technology for preparation of hydrogels has emerged as an exciting opportunity for tissue regeneration applications [35,36,37]. In this context, functionalized-starch able to photo-crosslink in water to be exploited as photo-curable and 3D-printable hydrogel precursor would be of high interest. Therefore, starch was methacrylated followed by evaluation of light processability by photo-curing in a mold or by digital light processing (DLP) 3D printing. Furthermore, the light processed hydrogels were characterized for rheological and mechanical properties and cytotoxicity.

## 2. Materials and Methods

### 2.1. Materials

High-amylose Hylon VII maize starch (70% amylose) was obtained from Ingredion, Goole, UK. Methacrylic anhydride (MA), triethylamine (TEA) (>99%), dimethyl sulfoxide (DMSO) (ACS reagent P99.9%), ethanol absolute, and bis(acyl)phosphane oxi lithium phenyl-2,4,6-trimethylbenzoyl phosphinate (LAP) were purchased from Sigma Aldrich (Milan, Italy).

### 2.2. Synthesis of Methacrylated Starch

High amylose maize starch and DMSO were placed in a round-bottom flask under stirring condition (6 g of maize starch in 200 mL DMSO) and the temperature was increased to 70 °C. After 30 min the starch had been gelatinized and the solution was cooled down to room temperature. Methacrylic anhydride was then added dropwise with a syringe and triethylamine, as a nucleophilic catalyst, was added slowly (molar ratio AGU:MA:TEA of 1:2:0.04). The final solution was kept at room temperature for 18 h. After 18 h the product was precipitated in ethanol, dissolved in deionized water and again precipitated in ethanol. This procedure was repeated two times in order to purify the product. The final aqueous solution was lyophilized.

### 2.3. Photocuring of Methacrylated Starch

To fabricate starch hydrogels, aqueous solutions containing 5, 10, or 15 wt% of functionalized starch were prepared. Testing of higher concentration was not possible due to solubility limit of MA-starch in water. The photo-initiator selected was lithium phenyl-2,4,6-trimethylbenzoylphosphinate (LAP), which was added in a content of 1 wt% with respect to the starch weight. The photocurable formulations were then placed in a silicon mold and cured for 1 min under UV-DIMAX lamp with a light intensity of 115 mW/cm^2^. The formed hydrogels were named according to the original starch content, i.e., MA-Starch5, MA-Starch10, and MA-Starch15, for the hydrogel prepared from aqueous solution with 5, 10, and 15 wt% of starch, respectively.

### 2.4. 3D-Printing of Methacrylated Starch

The MA-Starch10 formulation (water-based formulation containing 10 wt% of functionalized starch and 1 wt% of LAP photo-initiator) was 3D printed using a lithography-based ASIGA UV-MAX DLP printer (Robot Factory S.r.l., Venice, Italy). The DLP printer had a light emission at λ = 385 nm and a XY pixel resolution of 62 μm. Approximately 6 mL of starch–water solution was placed in the printer vat. The UV light intensity was set to 10 mW/cm^2^ with an exposure time of 5 s for a layer thickness of 50 µm.

### 2.5. Nuclear Magnetic Resonance (NMR)

Starch and MA-Starch were analyzed by Bruker Advance 400 Fourier Transform NMR spectrometer (FT NMR, Bruker, Billerica, MA, USA) operating at 400 MHz. The ^1^H-NMR was conducted at room temperature. Approximately 7 mg of each sample were dissolved in 1 mL of DMSO-d6. The ^13^C-NMR was conducted at 60 °C with 20 mg/mL as starch concentration in DMSO-d6.

### 2.6. Attenuated Total Reflectance-Fourier Transform Infrared Spectroscopy (ATR-FTIR)

The FTIR spectra of starch, MA-starch and the crosslinked hydrogels were recorded by Perkin Elmer Spectrum 2000 FTIR spectrometer (Perkin Elmer, Norwalk, CT, USA) equipped with a single reflection attenuated total reflectance (ATR) accessory (golden gate). The samples were scanned from 4000 to 500 cm^−1^ with 4 cm^−1^ resolution.

### 2.7. Photorheology and Rheology

The tests were performed with an Anton PAAR Modular Compact Rheometer (Physica MCR 302, Graz, Austria) using a parallel plate configuration (φ = 15 mm) with a quartz bottom glass. The gap value was set as 300 μm. All experiments were carried out at T = 25 °C. All rheological measurements were performed in triplicate. Oscillatory rheometer operating in time, stress and frequency sweep modes was used to monitor the viscoelastic properties associated with the crosslinking kinetics and to characterize the mechanical properties of the photo-crosslinked networks. The time sweep experiment was performed in the linear viscoelastic region (LVR) at a constant strain amplitude (γ) of 0.5% and a constant frequency (ω) of 6 rad/s to monitor the in-situ gel formation by following the evolution of elastic storage modulus G’ with time. After 30 s, the UV lamp was switched on.

The UV lamp used in the photo-rheology experiments was a Hamamatsu LC8 lamp (Hamamatsu City, Japan) with a light intensity of 28 mW/cm^−2^. The samples were irradiated from the bottom. An amplitude sweep experiment was also performed on the cured hydrogels γ [0.01–100%] at constant ω = 6 rad/s. After that, a frequency sweep experiment was performed on a freshly prepared sample; ω [0.1–100 rad/s] with a constant γ = 1%. It was possible to determine the crosslinked structure parameters using the storage modulus in the frequency independent plateau region (G’_P_). In this plateau, the energy applied is stored elastically by the polymer segments between two crosslinks that behave as entropic springs. In fact, the polymer chains are not capable of sliding by one another since the stress velocity is higher than the segment relaxation ability. The network parameters crosslink density (ν_e_) and distance between two entanglement points (ξ) were calculated by Equations (1) and (2), respectively.

(1) $$\nu_{e}=\frac{G_{P}^{’}N_{A}}{\mathrm{RT}}\;,$$

(2) $$\xi =\frac{1}{\sqrt[{3}]{\nu_{e}}},$$

R is the universal gas constant, T is the temperature, and N_A_ is the Avogadro’s number [38].

### 2.8. Compression Test

The compression tests were performed with MTS QTest^TM^/10 Elite controller using TestWorks^®^ 4 software (MTS Systems Corporation, Edan Prairie, Minnesota, USA) The unconfined uniaxial compression was performed with cell loaded of 10 N and a test speed of 0.5 mm/min. The samples tested had cylindrical geometry: φ = 10 mm X h = 11 mm. The data acquisition rate was set as 20 Hz. The compressive modulus was calculated by TestWorks 4 software. The modulus was estimated as the slope of the linear region of the stress–strain curves.

### 2.9. Swelling Behavior

The swelling behavior was studied by means of conventional gravimetric procedure. Air-dried hydrogels were placed in deionized water at 25 °C. The weight increase was monitored during different time-intervals by taking the samples from the aqueous media and weighting them after the removal of free water on the surface with filter paper. The swelling ratio percentage (S_w%_), the equilibrium swelling ratio (S_eq_), and the equilibrium water content (EWC) were calculated with Equations (3)–(5), respectively [39,40].

(3) $$S_{w\%}=\frac{\left(W_{s}-W_{d}\right)}{W_{d}}*100,\;$$

(4) $$S_{eq}=\frac{W_{eq}-W_{d}}{W_{d}},$$

(5) $${\mathrm{EWC}}_{\%}=\frac{W_{\mathrm{eq}}-W_{d}}{W_{\mathrm{eq}}}*100,$$

where W_s_, W_d_, and W_eq_ are the weight of the sample in the swollen, dry, and swollen at equilibrium states, respectively.

### 2.10. Gel Content

The gel content percentage (G%) was calculated according to Equation (6):

(6) $$G\%=\frac{W_{1}}{W_{o}}*100,$$

where W_1_ is the weight of the dry gel after the treatment with deionized water and W_0_ is the weight of dry sample before the treatment.

### 2.11. Differential Scanning Calorimetry (DSC)

Differential scanning calorimetry was performed with a Mettler-Toledo DSC 820 (Mettler-Toledo, Stockholm, Sweden). Samples having masses of approximately 7 mg were inserted in 100 μL aluminum pans with pierced lids. The applied heating rate was 10 °C min^−1^ in a nitrogen atmosphere (rate 50 µL/min). Thermal behavior of the samples was investigated using the following heating–cooling cycles: the temperature was raised from room temperature to 100 °C, then cooled down to −30 °C, and raised again to 200 °C. The glass transition temperature (T_g_) was determined from the second heating curve.

### 2.12. Cell Viability

Cell viability assay was performed to evaluate the effect of the degradation products of MA-Starch hydrogels on cell viability. BJ human fibroblast cells (ATCC^®^ CRL-2522™) were cultured on three different 96-well plates at a cell density of 2 × 10^4^ cells/well for 24 h to reach confluence using DMEM-Dulbecco’s Modified Eagle Medium enriched with 10% fetal bovine serum and 1% penicillin/streptomycin (Carlo Erba, Milan, Italy). Simultaneously, three hydrogels prepared with three different methacrylated starch concentrations (5, 10, and 15 wt%) were soaked for 24 h in 1 mL of DMEM. In each case, 0.1 g of hydrogel was soaked in 1 mL of the medium. After 24 h, the media were collected and filtered through 0.22 µm filters to guarantee sterility. Then, the culture medium was removed from each well of confluent cells and substituted with the supernatant collected from the hydrogels. Controls (CTRL) were obtained using normal medium. After 24 h of incubation, the supernatant was carefully removed and the cell viability assay was performed using non-fluorescent resazurin, which is converted into a highly red fluorescent dye (resorufin) by cell metabolism. Briefly, a volume of 100 µL of 0.1 mg/mL resazurin solution, obtained by diluting a resazurin working solution (1 mg/mL in phosphate buffered saline-PBS, Sigma Aldrich, Milan, Italy) into DMEM, was added in each well and the cultures were incubated for 1 h at 37 °C. Then, the fluorescent signal was monitored at 530 nm excitation wavelength and 590 nm emission wavelength using a plate reader (Victor X3, Perkin Elmer, Milan, Italy). Cell viability was calculated as a percentage value compared to CTRL. Six samples for each condition were used and the experiments were performed three times. GraphPad Prism^®^ software was used for one or two analysis of variance (ANOVA). Values * *p* < 0.05, ** *p* < 0.01, *** *p* < 0.001 were considered statistically significant.

## 3. Results and Discussion

**Scheme 1:**
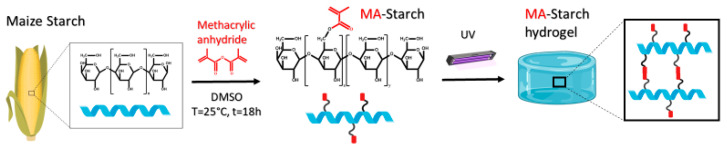
Schematic illustration of starch methacrylation and photo-crosslinking reaction. The structure presented for MA-Starch presents the most likely structure where some of the primary alcohol groups were methacrylated.

Starch as a raw material for light processable hydrogels was evaluated. To render light processability, starch was first methacrylated (Scheme 1). The methacrylated starch (MA-Starch) was then evaluated for photocuring reactivity, and DLP 3D printability, followed by establishment of the properties of the light processed hydrogels including mechanical and rheological testing and cell viability.

### 3.1. Confirmation of Starch Methacrylation

**Figure 1:**
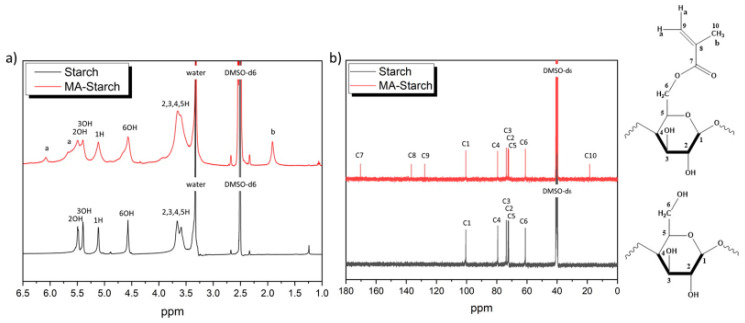
(**a**) ^1^H-NMR spectra; and (**b**) ^13^C-NMR spectra for Starch-reference before methacrylation and methacrylated MA-Starch with peak assignments.

The methacrylation reaction was evaluated and confirmed by ^1^H-NMR, ^13^C-NMR, and FTIR spectroscopy. Firstly, the ^1^H-NMR spectrum (Figure 1) recorded for Starch-reference, treated with the same process but without methacrylation, shows typical starch peaks: δ = 3.59 and δ = 3.66 represent the CH protons at 2, 3, 4, and 5 positions; the peak at δ = 5.11 represents the C1 anhydroglucose carbon; and δ = 4.57, δ = 5.40, and δ = 5.49 represent the OH protons at 2, 3, and 6 positions, respectively. The huge peak at δ = 3.35 is due to the inevitably absorbed water in the starch structure [41]. The ^1^H NMR spectrum recorded for MA-Starch shows the presence of characteristic peaks that correspond to the protons of methacrylic double bond = CH_2_ (δ 5.66−6.07 ppm) and methyl groups -CH_3_ (δ 1.9 ppm). These spectral changes are consistent with the structural modification of starch. However, as shown in Figure 1a, the peaks of the MA-Starch spectrum are broader compared to Starch-reference. This can be explained by the partial methacrylation of the hydroxyl-groups in the starch chains, leading to different structures due to mixture of free and methacrylated alcohol groups in the repeating units.

The degree of substitution (DOS) for the hydroxyl groups was obtained as the ratio of integrals of the ^1^H-NMR peaks using Equation (7):

(7) $$\mathrm{DOS}=\left(\frac{\frac{I_{1.9}}{3}}{I_{5.11}}\right)/3,$$

where I_1.9_ and I_5.11_ are the integral intensity of CH_3_ protons (named b in the spectra) and the proton of the anhydrous glucose unit (AGU) unit in α-carbon (1H position). The formula is divided by three since there are three -OH groups in each AGU. The degree of substitution obtained was 0.08 (I_1.9_ ≈ 0.75, I_5.11_ ≈ 1), which can be translated into one methacrylated alcohol-group in every fourth glucose ring (Figure 1). This degree of substitution is very similar to what was reported for starch methacrylated with glycidyl methacrylate [29].

The ^13^C-NMR spectra of Starch-reference and MA-Starch are shown in Figure 1b. All the carbon atoms of starch repeating unit can be detected and assigned. The signals at δ = 100.54 ppm, δ = 79.37 ppm, δ = 73.74 ppm, δ = 72.55 ppm, δ = 72.12 ppm, and δ = 61.09 ppm in the Starch-reference spectrum can be attributed to C1, C4, C3, C2, C5, and C6 of the glucose unit, respectively. In the ^13^C-NMR spectrum of MA-Starch, there are additional signals from the methacryl-groups. These are seen at δ = 18.45 ppm (methyl carbon), δ = 136.61 ppm and δ = 127.75 ppm (double bond carbons) and δ = 170.38 ppm (C=O carbon).

**Figure 2:**
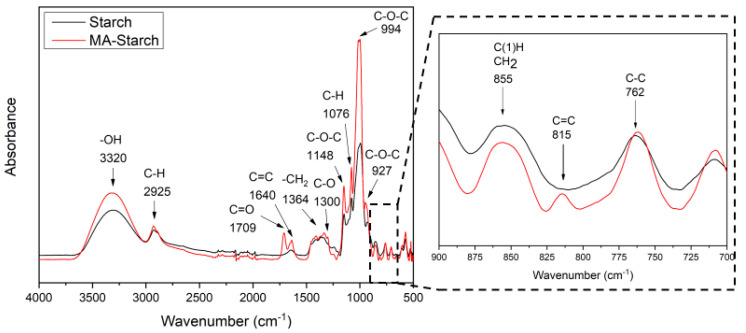
FTIR spectra of Starch-reference recorded before (black curve) and MA-Starch after the methacrylation reaction (red curve).

The methacrylation reaction was further confirmed by FTIR. Figure 2 presents the FTIR spectra of Starch-reference and MA-starch. The FTIR spectrum of Starch-reference displays its characteristic functional groups. The broad peak at 3320 cm^−1^ is attribute to the -OH vibration. The peak at 2925 cm^−1^ is the -CH_2_- stretching vibration. The band at 1638 cm^−1^ is the deformation vibration of the hydroxyl group of H_2_O absorbed in the amorphous regions of starch. The peak at 1148 cm^−1^ is the C-O-C symmetric stretching of cyclic ether group, while the peak at 1076 cm^−1^ is the C-H bending vibration. The peaks at 994 and 927 cm^−1^ are assigned to the stretching and skeletal vibration of the C-O-C α-1-4 glycosidic linkage, respectively. Finally, the peaks at 855 and 762 cm^−1^ can be assigned to the C(1)-H, CH_2_ deformation and C-C stretching vibration, respectively [42,43]. In the FTIR spectrum of MA-Starch, it is possible to observe four new peaks that strongly support a successful methacrylation of some of the starch hydroxyl groups. The peaks at 1709 and 1300 cm^−1^ can be attributed to the C=O and C-O stretching vibrations, respectively, while the two peaks at 1640 and 815 cm^−1^ are assigned for the C=CH_2_ out of plane bending vibrations [28,44,45].

### 3.2. Photo-Rheology of the Photo-Crosslinking Reaction for Forming Starch Hydrogels

**Figure 3:**
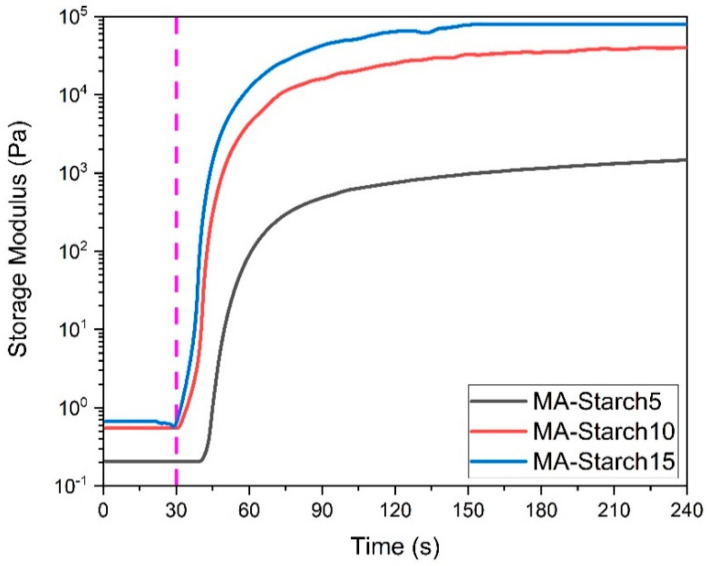
Photorheological evaluation: Elastic modulus G’ as a function of time for the photocurable starch formulations at different concentrations of MA-starch. The lamp was switched on after 30 s (the dashed line).

The methacrylated-starch was dispersed in water with LAP photo-initiator and the rheological properties of the starch/water/LAP formulations were recorded during the photo-crosslinking reaction to evaluate the reaction kinetics. Initially, an amplitude sweep experiment was performed on the liquid formulations to determine the linear viscoelastic region (LVR). The reaction is considered as completed when a G’ plateau is reached. Figure 3 shows that the formulations with 10 and 15 wt% starch started reacting immediately and reached a G’ plateau after 90 s, while the 5 wt% starch formulation showed a delay in the curing process and reached a G’ plateau after 120 s. This is probably explained by the low starch concentration in water, which in combination with the overall low DOS decreased the velocity of the radical reaction.

### 3.3. Characterization of the Photo-Crosslinked Starch Hydrogels

**Figure 4:**
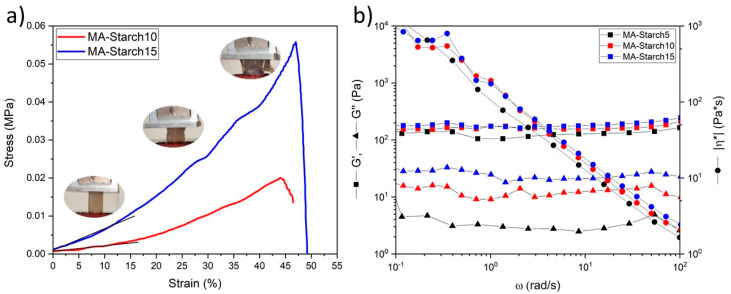
(**a**) Stress–strain curves of MA-Starch 10% and 15%; and (**b**) storage modulus G’, loss modulus G’’, and the dynamic viscosity |η*| as a function of the applied frequency ω for the MA-Starch hydrogels.

The photocured hydrogels were fully characterized for their mechanical, rheological, and swelling properties. The compressive stress–strain curves of MA-Starch10 and MA-Starch15 photocured cast discs are shown in Figure 4a. The compressive Young’s modulus values (E_c_) are reported in Table 1. The simple increase of the MA-Starch concentration in water from 10 to 15 wt% increased the compressive stiffness of the hydrogels from 13 to 20 kPa. These values are similar to the ones reported for photo-crosslinked hemicellulose and silk fibroin networks applied for tissue engineering [46,47]. This range also covers the stiffness of different native body tissues, which is promising for utilization of the hydrogels in tissue engineering applications, where the cell microenvironment can be mimicked by matching the stiffness of different body tissues [48].

For rheological evaluation, the polymer solutions (300 μL) were crosslinked directly on the parallel plate for instant measurements. To determine the linear viscoelastic region of the hydrogels, an amplitude sweep experiment was initially performed. After that, a frequency sweep experiment was performed on a freshly prepared sample with a strain amplitude of 1%. The results of the frequency sweep experiment, including G’, G’’, and |η*|, are shown in Figure 4b.

The complex viscosities of the three hydrogels have approximately the same slope of −0.9, which means that all the networks exhibit a pseudo-plastic behavior [38]. All the hydrogels also show linear and stable behavior for G’ and G’’ values in the ω range tested. Their progressions are parallel, but the G” values lie in one order of magnitude lower region. This means that the elastic component of the material is dominant over the viscous behavior, which is common for different gel structures [49]. The values of G’ and G’’ are also comparable with the ones obtained in a previous work for carboxymethylated starch hydrogels [50,51]. The network parameters were evaluated on the G’_p_ at ω = 10 rad/s and collected in Table 1. Increasing the MA-Starch concentration decreased the distance between two entanglements points and increased the crosslink density.

**Figure 5:**
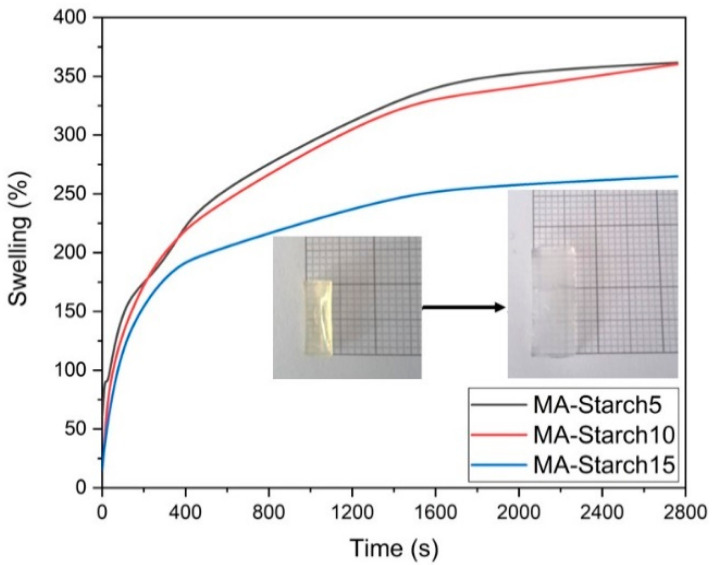
Swelling behavior of the three MA-Starch derived hydrogel formulations.

Figure 5 reports the swelling curves for the three hydrogels in water, while Table 1 collects the swelling at equilibrium Seq, equilibrium water content EWC, and gel percentage G% values for the three hydrogel formulations. As seen in the graph, the swelling capability of MA-Starch5 and MA-Starch10 were very similar, while the swelling capability of MA-Starch15 was lower. However, the values obtained from MA-Starch5 does not corresponds to the maximum swelling capability but only to the maximum swelling before breaking. The values of MA-Starch10 and MA-Starch15 are in good agreement with the values obtained from the rheological measurement on the crosslink density, as higher crosslink density leads to lower swelling degree. The gel content of the dried samples was measured as described in the Experimental Section 2.10 and it reached 100% for all three formulations (Table 1). Starch-reference, MA-starch and the three MA-starch hydrogels (5, 10, and 15%wt of MA-starch) were analyzed via differential scanning calorimetry analysis to investigate the glass transition temperatures (T_g_) (see Table 1). There was no significant difference between the T_g_ of the methacrylated starch and the T_g_ after photo-crosslinking of the materials.

### 3.4. Cell Viability of the Photo-Crosslinked Starch Hydrogels

**Figure 6:**
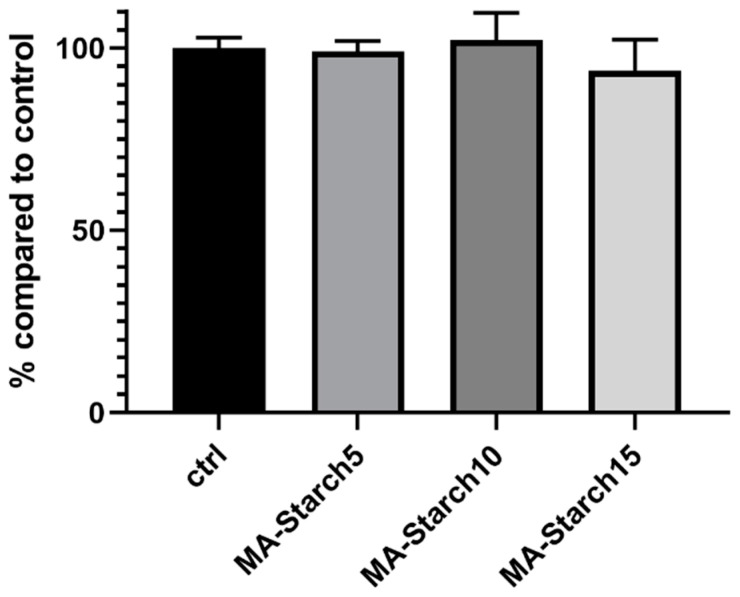
Cell viability of BJ cells culture using the supernatants collected from the starch-based hydrogels compared to the control conditions (black bars).

Human fibroblast cells were cultured on the supernatants from the three hydrogel formulations. Starch has previously been utilized in many biomedical applications for fabrication of scaffolds, hydrogels, and drug delivery systems [52,53] showing a good biocompatibility. The starch content in the present study had no negative effects on the cell viability (Figure 6). The cell viability of all three formulations was also very similar to the control, indicating no negative effects from the methacrylation, photo-crosslinking reaction and LAP photo-initiator. These first results indicate that the prepared starch-based photo-crosslinked hydrogels could have potential in biomedical applications, such as tissue engineering and also as cell carriers through dispersion of cells into the photocurable formulations.

### 3.5. The 3D Printability of the Methacrylated Starch Hydrogels by DLP

**Figure 7:**
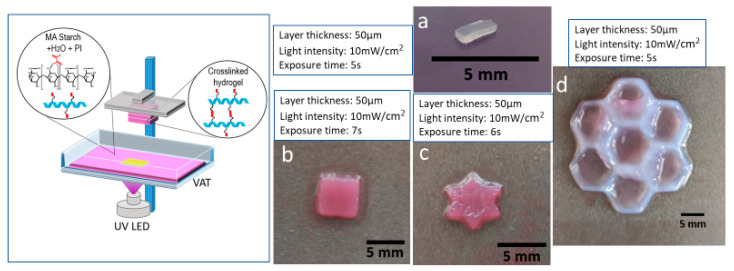
3D DLP printed hydrogels: (**a**) smallest hydrogel printed without the use of dye [1.5 mm × 0.5 mm × 2 mm]; (**b**,**c**) simple geometries [5 mm × 5 mm × 1 mm]; and (**d**) complex flower geometry [24 mm × 24 mm × 4 mm].

The fast photo-curing reactivity of the formulations and the obtained mechanical properties indicated that the MA-Starch could be suitable photo-curable resin for the vat 3D printing technology [54]. The 3D printability of the methacrylated starch by DLP was therefore evaluated. The formulation with 10 wt% MA-Starch was selected since the hydrogel prepared with this concentration showed a compression Young’s modulus (E_c_ = 13 kPa) close to the elasticity of muscle tissue (12 kPa) [48]. Simple geometries were designed by CAD and printed in order to evaluate the light processability of the bio-based formulation by DLP printing and to investigate the achievable resolution. The common photocurable 3D printable formulations usually exploit a dye or colorant to avoid the light diffusion in the vat to achieve a better resolution [55]. In the present case, different tests were performed. First, neat formulation without the addition of any dye was tested giving promising results with good resolution on the x-y plane. The smallest feature that could be obtained had dimensions of 1.5 mm × 0.5 mm × 2 mm, presenting sharp edges and limited over-polymerization in the vat (Figure 7a). Subsequently, the addition of a methyl red dye (0.2 wt% based on the weight of the dry starch) was evaluated (Figure 7b,c). The addition of the dye implied the need of changing the printing parameters. Several trials were performed to achieve lower over-polymerization along the profile of the printed shapes. However, since the cytotoxicity of methyl red was not assessed, other alternatives should be searched for. The cell viability test showed that the pH of the starch solution in the cell medium was neutral, which led to a solution with pink color. This suggested the possibility to substitute the aqueous dye solution with DMEM solution in the 3D printable MA-starch formulation. A honeycomb structure was then designed and successfully printed from the DMEM containing formulation, showing that millimeter-thick wall structures could be built from this formulation (Figure 7d). The use of such a light-dye led to a certain degree of over polymerization in the x-y plane, demonstrating the need to correctly balance the different ingredients and printing parameters for further printing studies. All the printed objects required a post-curing process, which was regulated with the help of the photo-rheology test to achieve complete crosslinking reaction. All the 3D printed hydrogels were mechanically stable for easy handling.

## 4. Conclusions

Methacrylated maize starch was successfully light processed to hydrogels either by photo-curing with UV-lamp or by DLP 3D-printing directly from aqueous solution. First, starch was methacrylated and the reaction was confirmed by ^1^H-NMR, ^13^C-NMR, and FTIR spectroscopy. The methacrylated starch was dispersed in water at different concentration in the presence of LAP photo-initiator. Photo-rheology confirmed rapid in-situ gel formation by following the evolution of elastic storage modulus G’ with time. The measured G’ plateau values were 1.5, 39.9, and 67.6 kPa for the hydrogels obtained in the presence of 5, 10, and 15 wt% of MA-starch. These values are comparable with other methacrylated biobased hydrogel systems reported in the literature. Rheological characterization of the crosslinked materials was performed and the network parameters were evaluated on the G’_p_ at ω = 10 rad/s. Increasing the MA-Starch concentration increased the crosslink density, which led to decreased swelling capability. The supernatants of the methacrylated starch-based photo-crosslinked hydrogels did not exhibit cytotoxicity against human BJ fibroblast cells in any of the three tested hydrogel concentrations. Finally, the 3D printability of 10 wt% MA-Starch was successfully demonstrated. These findings are promising for future applications of light processed starch in tissue engineering and as cell carriers.

## Figures and Tables

**Table 1:** Properties of Starch-reference, MA-Starch, and MA-Starch hydrogels.

Hydrogel	Starch	MA-Starch	MA-Starch5	MA-Starch10	MA-Starch15
**E_c_ [kPa]**	-	-	-	12.7 ± 0.4	20.3 ± 1.7
**G’_P_ [Pa]**	-	-	105	159	178
**ν_e_ [m^−3^]**	-	-	2.55 × 10^22^	3.86 × 10^22^	4.32 × 10^22^
**ξ [m]**	-	-	3.40 × 10^−8^	2.96 × 10^−8^	2.85 × 10^−8^
**S_eq_**	-	-	3.6 *	2.7	3.6
**EWC%**	-	-	78 *	78	73
**G%**	-	-	100%	100%	100%
**T_g_ (°C)**	105 ± 2	115 ± 1	106 ± 2	106 ± 9	101 ± 8

* Maximum value obtained before the sample failure. Ec, compressive Young’s module values; ν_e_, crosslink density; ξ, distance between two entanglements points; S_eq_, swelling at equilibrium; EWC, equilibrium water content; G%, gel percentage; T_g_, glass transition temperature.
